# Polyamine-Induced Rapid Root Abscission in *Azolla pinnata*

**DOI:** 10.1155/2012/493209

**Published:** 2012-09-11

**Authors:** Sushma Gurung, Michael F. Cohen, Jon Fukuto, Hideo Yamasaki

**Affiliations:** ^1^Faculty of Science, University of the Ryukyus, Nishihara 903-0213, Japan; ^2^Department of Biology, Sonoma State University, Rohnert Park, CA 94928, USA; ^3^Department of Chemistry, Sonoma State University, Rohnert Park, CA 94928, USA

## Abstract

Floating ferns of the genus *Azolla* detach their roots under stress conditions, a unique adaptive response termed rapid root abscission. We found that *Azolla pinnata* plants exhibited dose-dependent rapid root abscission in response to the polyamines spermidine and spermine after a substantial time lag (>20 min). The duration of the time lag decreased in response to high pH and high temperature whereas high light intensity increased the time lag and markedly lowered the rate of abscission. The oxidation products of polyamines, 1,3-diaminopropane, **β**-alanine and hydrogen peroxide all failed to initiate root abscission, and hydroxyethyl hydrazine, an inhibitor of polyamine oxidase, did not inhibit spermine-induced root abscission. Exposure of *A. pinnata* to the polyamines did not result in detectable release of NO and did not affect nitrite-dependent NO production. The finding of polyamine-induced rapid root abscission provides a facile assay for further study of the mode of action of polyamines in plant stress responses.

## 1. Introduction

Polyamines (PAs) are small positively charged aliphatic molecules ubiquitous in almost all life forms. In plants, spermine (Spm), spermidine (Spd), and their precursor putrescine (Put) are the major PAs present in cells at micromolar-to-millimolar concentrations [1]. PAs have been implicated in a wide range of life processes in plants including seed germination, growth, floral initiation, floral development, pathogen defenses, and environmental stress responses [2–4]. Following the classic report of Richards and Coleman [5] on PA accumulation in potassium starved leaves, many investigations have demonstrated the physiological relevance of PAs in response to diverse environmental stresses, including heavy metal stress, SO_2_ pollution, osmotic stress, chilling stress, drought stress, pH stress, nutritional stress, biotic stress, and heat stress (reviewed by [4, 6–8]). Although the explicit physiological role of the increase in PA remains obscure, exogenous addition of PAs to plants under stress conditions has been reported to alleviate stress damage or to increase tolerance to adverse environments [6, 8]. A recent study suggested crosstalk with or direct involvement of the similarly multifunctional molecule nitric oxide (NO) in a PA-mediated response [9]. Regardless of the fact that underlying mechanism of its action remains unclear, it is evident that PAs are an integral part of plant stress responses.

Plants, owing to their sessile nature, are compelled to endure stress due to perpetual environmental changes. They do so through morphological, biochemical, or physiological adjustments. Under stressful conditions, the tiny globally-distributed water ferns of the genus *Azolla *[10] respond through a unique rapid loss of their roots, a phenomenon termed rapid root abscission [11–13]. Such shedding sets its fronds free from root-entangled mats and facilitates their dispersion to a potentially better environment. The phenomenon is thus considered to be an important survival strategy for *Azolla *[12, 13]. 

Uehda and coworkers have demonstrated that rapid root abscission is primarily due to rapid osmotic expansion of cells at the base of the roots with presumptive activation of hydrolytic enzymes in the cell wall to hasten the root separation [12, 14]. Unlike typical abscission events in other plants, programmed cell death does not appear to be involved in the process. 

Previous studies revealed that rapid abscission in *Azolla *can artificially be induced by nutrient stress, a high concentration of nitrite [11], chemical stress (i.e., treatment with the inhibitors of oxidative phosphorylation sodium azide, 2, 4-nitrophenol and carbonyl cyanide *m-*chlorophenyl-hydrazone) [12], and by transient exposure to high temperature [13]. An array of stimuli culminating to the same response in *Azolla* leave open the possibility of a common inducer or internal mediator involved in the root abscission process. 

Since higher plants exposed to a broad spectrum of abiotic stresses exhibit alteration in PA metabolism, we sought to investigate the potential role of PAs in rapid root abscission of *Azolla*. Alterations of PA content have been observed during high-density induced sporulation in *Azolla* [15] and in response to exposure to ozone [16] and nitrogen dioxide [17]. To the best of our knowledge, no study has yet specifically examined the effect of polyamines on rapid root abscission. The aim of this study was to assess the effect of exogenously applied PAs on rapid root abscission phenomenon. Here we report that PAs are potent inducers of rapid root abscission in *Azolla*.

## 2. Materials and Methods

### 2.1. Plant Material

Laboratory cultures of *Azolla pinnata* were used for the study. Fronds of the water fern were originally collected from a local taro field in Okinawa, Japan. The plants were thoroughly washed to remove attached mud and debris. Surface disinfection was done according to the method described by Gerald and Berger [18]. Fronds were treated with a solution containing 0.12% (v/v) sodium hypochlorite and 0.01% (v/v) Triton X-100 for 3 min and then repeatedly washed in a large volume of distilled water before transferring to the nutrient medium.

### 2.2. Nutrient Medium and Culture Conditions

A two-fifth strength nitrogen-source-free Hoagland's E-medium [19] was used for the culture of *A. pinnata*. It contained 200 *μ*M potassium dihydrogenphosphate, 400 *μ*M magnesium sulphate heptahydrate, 0.31 *μ*M zinc sulphate heptahydrate, 0.14 *μ*M copper sulphate pentahydrate, 14.32 *μ*M ferric chloride hexahydrate, 920 *μ*M calcium chloride, 3.68 *μ*M manganese chloride tetrahydrate, 18.5 *μ*M boric acid, 0.15 *μ*M disodium molybdate (VI) dehydrate, and 41.26 *μ*M ethylene diamine-N, N, N′, N′-tetraacetic acid, disodium salt, dehydrate. In addition, 0.12 *μ*M cobalt chloride hexahydrate [20] was also supplied into the medium, and pH was adjusted to 5.8 with potassium hydroxide. Fronds were grown in a plant growth chamber (Type FLI-2000H, Eyla, Japan) maintained at 27 ± 1°C, 80% humidity, 16 : 8 h light : dark photoperiod and 50 *μ*mol m^−2^ s^−1^ (at plant level) provided by fluorescent lamps (Type FL 40 SBR-A, NEC, Japan). 

For experiments, 15–20 fronds were randomly selected from the cultured *A. pinnata* stock and derooted manually using forceps. Rootless fronds were then washed at least twice in distilled water and cultured in the nutrient medium. The fronds were transferred to a fresh medium every four days. Abscission experiments were carried out with fronds which had produced new roots of the same age.

### 2.3. Polyamine Treatments

In order to assess the potential of PAs to induce root abscission, 2-3 fronds (20–30 roots) of *A. pinnata* were first placed on a 10 mM potassium phosphate buffer solution, pH 7 (control set) at a room temperature (25°C). Then PA putrescine dihydrochloride, spermidine or spermine (0.5–5 mM) was subsequently added. The number of roots (>10 mm in length) abscised was recorded every 10 min for 2.5 hr. Total abscission rate (%) was determined as the ratio of the detached to the initial number of roots.

### 2.4. Effect of pH on PA-Induced Abscission

Exogenous addition of 2 mM Spm and Spd resulted in change in the pH of the phosphate buffer rising from neutral to 9.0 and 8.3, respectively. In order to determine whether the pH rise influenced PA-induced abscission in *A*. *pinnata*, experiments were conducted with or without readjusting the pH of the buffer to 7.0 after addition of 2 mM Spm or Spd using hydrochloric acid. We also assessed root abscission in response to 2 mM spermine tetrahydrochloride (Spm·4HCl); the acidic form of Spm which does not increase the pH to alkaline. As a control, *A. pinnata* fronds were placed in 10 mM potassium phosphate buffer with pH ranging from 4 to 10 and root loss monitored for 2.5 hr. 

### 2.5. Effect of Temperature on PA-Induced Abscission

To study the effect of temperature, *A. pinnata* fronds were transferred to beakers containing neutral phosphate buffers, and then 2.0 mM Spm was exogenously added. The beakers were then placed in a water bath maintained at 25°C, 27°C, 32°C, and 38°C. Abscission rate was recorded every 10 min for 2 h.

### 2.6. Effect of Light on PA-Induced Abscission

In order to examine the effect of light and light intensity, *A. pinnata* fronds suspended in phosphate buffer containing 2 mM Spm were subjected to varying light environment, that is, dark condition (0.01 *μ*mol m^−2^ s^−1^), ambient light (5 *μ*mol m^−2^ s^−1^), and high light (250 *μ*mol m^−2^ s^−1^). Abscission was monitored every 10 min for 2 h.

### 2.7. PA Degradation Product Treatments

In order to gain some insight into the identity of the molecular inducer in PAs-induced abscission, experiments were performed using the catabolic products of higher PAs. Polyamine oxidase (PAO) degrades Spd and Spm and produces a stress signaling molecule hydrogen peroxide (H_2_O_2_) and 1, 3-diaminopropane (Dap) [21]; the latter can be further converted to *β*-alanine (Ala) [22]. *A*. *pinnata* fronds were treated with 2 mM Ala, Dap, or 2–10 mM H_2_O_2_ dissolved in the neutral buffer (pH 7.0), and the abscission induced by each treatment was monitored.

### 2.8. Measurement of NO to Determine the Effect of PAs on Nitrite-Dependent NO Production in *A. pinnata*

PAs are reported to stimulate NO production in plants via an unknown pathway [23, 24] as well as modulate the activity of nitrate reductase (NR) [25], the key enzyme in the nitrite-dependent NO production in plants. NO emission was detected with a Sievers Nitric Oxide Analyzer (NOA) 280i, which employs ozone-chemiluminescence technology, and the data was collected by NOAnalysis software. 

A confluent layer of *A. pinnata* covering the surface area of a 10 cm diameter petridish containing 20 mL of neutral phosphate buffer was used for NO measurement at room temperature (25°C). The petridish contained two holes, one at the side for the inserting the tip of NOA and the other at the top cover to supply the chemicals. To ensure proper mixing, apparatus was placed in shaker. Basal NO production of *A. pinnata* in neutral phosphate buffer was measured for 10 min. Then 0.1 mM sodium nitrite, a well-known NO source in plants [26], was supplied to initiate NO production. After 10 min, 2 mM of Spm or Spd was added to examine the effect of PAs on nitrite-dependent NO production. At 25 min, the same concentration of PA was added and the NO measurement carried out until 50 min. Spermidine trihydrochloride (Spd·3HCl) and spermine tetrahydrochloride (Spm·4HCl), the acidified form of Spd and Spm, respectively, were used to prevent the medium pH from increasing to highly alkaline.

### 2.9. Morphological Observations

Throughout the experimental period, digital photographs were taken at different intervals of time. Photos of entire fronds were taken with a digital camera (EOS kiss digital, Canon, Japan). For close-up observations, a zoom stereomicroscope (Model SZ 61, Olympus, Japan) with a digital camera (Model C7070WZ, Olympus, Japan) was used. The proximal portion of an abscised root was observed under a light microscope (Model Eclipse 80i, Nikon, Japan).

## 3. Results

### 3.1. PA-Induced Rapid Root Abscission in *A. pinnata*

**Figure 1:**
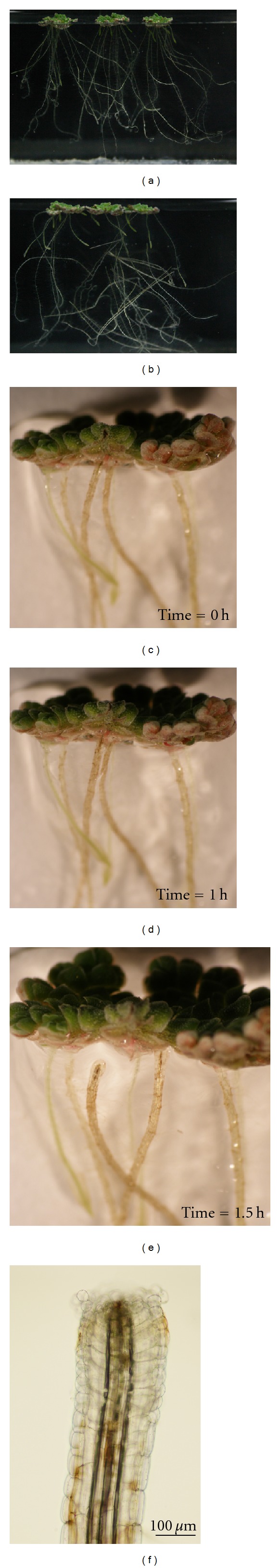
Photographs of polyamine-induced rapid root abscission in *A. pinnata*. Photos taken (a) before and (b) after the addition of 2 mM spermine. (c)–(e) Micrographs showing a sequential process of the root detachment. (f) Micrograph of an abscised root showing rounded cells at its proximal portion. Bar represents 100 *μ*m. The experimental conditions were similar to those in Figure 2.

Figure 1 presents a typical sequence of events of root abscission induced by PAs. Exogenous addition of Spd or Spm to the free-floating *A. pinnata* fronds caused significant shedding of roots within 2.5 hours after the treatment (Figures 1(a) and 1(b)). The detached roots showed characteristic morphological changes as shown in Figures 1(c)–1(e). We observed that PA-induced abscission was accompanied by rounding off of the cells at the proximal end of the detached roots (Figure 1(f)), similar to that observed in response to inhibitors of oxidative phosphorylation [12]. 

### 3.2. PA Concentration-Dependent Rate and Onset of Root Abscission

**Figure 2:**
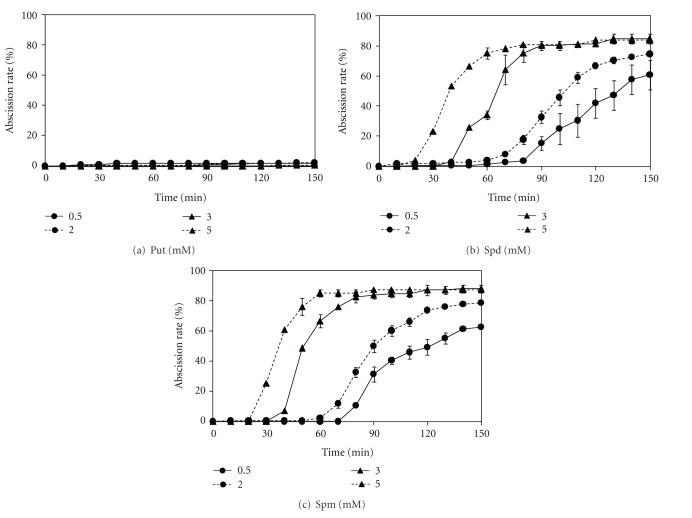
Effect of polyamines on root abscission in *A. pinnata*. Root abscission is represented as % of the initial number of roots following addition of individual polyamines at time zero. (a) Experiments in the presence of varying concentration of putrescine (0.5, 3 and 5 mM (*n* = 5) and 2 mM (*n* = 18)); (b) in the presence of varying concentration of spermidine (0.5 mM (*n* = 5); 2 mM (*n* = 17), 3 and 5 mM (*n* = 3)); (c) in the varying concentration of spermine (0.5, 3 and 5 mM (*n* = 3) and 2 mM (*n* = 19)). The standard error bars are only shown where larger than the symbols used. Assays were conducted in 10 mM potassium phosphate solution (pH 7).

Figure 2 shows time courses of root abscission in *A. pinnata* in response to 0.5–5 mM PAs. Spd or Spm induced significant rapid root abscission of *A. pinnata. *Both the extent of the response and the onset of abscission were dependent on the concentration of the PA. An increase in Spd or Spm concentration from 0.5 mM to 5 mM reduced the time for the onset of abscission from approximately 80 min to 30 min, respectively. Put showed no effect over the same time period. However, with a longer incubation period (48 hrs), Put was found to have the same effect as Spd and Spm, consistent with its conversion (albeit slow) to Spd and Spm (data not shown). Abscission rates in response to Spd and Spm showed similar concentration dependency within the 2.5 h experimental period (Figure 2). Neutral phosphate buffer alone without any added PAs failed to induce abscission (data not shown). The total abscission rate after 2.5 hr of 0.5–5 mM Put treatments was negligible (0.5–2%) (Figure 2(a)). There was no significant difference between the total abscission rates by 0.5 or 2 mM Spd and Spm. Moreover, an increase in their concentration to 3 or 5 mM resulted in only a 9-10% rise in the abscission rate (Figures 2(b) and 2(c)). Since Spm was the most effective PA, 2 mM Spm was used for further experiments.

### 3.3. Effects of pH on PA-Induced Root Abscission

**Figure 3:**
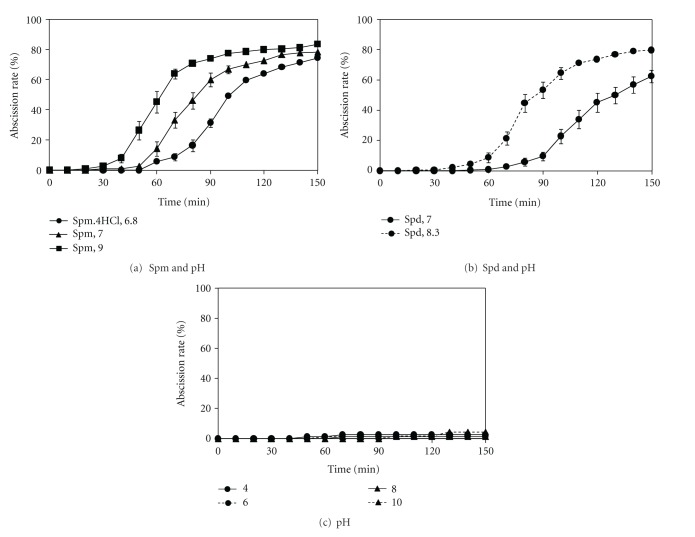
Effect of pH on PA-induced root abscission in *A. pinnata*. Root abscission is represented as % of the initial number of roots. Assays were conducted in (a) neutral phosphate buffer in the presence of 2 mM spermine at pH 9 and pH 7 (*n* = 10) and 2 mM spermine tetrahydrochloride (Spm·4HCl) at pH 6.8 (*n* = 3), (b) neutral phosphate buffer in the presence of 2 mM spermidine at pH 8.3 (*n* = 13) and pH 7 (*n* = 15), and (c) 10 mM potassium phosphate solution pH 4, 5, 6, 7, 8 (*n* = 3), 9 and 10 (*n* = 4). Hydrochloric acid was used to adjust the pH. In (c), since the results were identical, four representative data (pH 4, 6, 8, and 10) are shown for simplification. Standard error bars are only shown where larger than the symbols used.

It was suggested that enzyme(s) involved in the cell wall dissolution process during rapid root abscission in *Azolla *may be activated by neutral or weakly alkaline pH [14]. In the present study, we did observe substantial pH increases in the phosphate buffer solution upon addition of PAs and found that this pH increase did enhance the abscission response (Figure 3). However, an increase in pH could not solely be responsible for the PA-induced root abscission based on our observations of effective abscission in response to PAs even after readjustment of pH to 7 or acidified for Spm and an almost negligible occurrence of abscission in potassium phosphate buffers at various pHs ranging from 4 to 10 (Figure 3). We further verified pH effects with Good buffers (MES, MOPS, Hepes, Tricine) from pH 6 to pH 9 and confirmed that pH changes alone were ineffective in triggering the response (data not shown). 

### 3.4. Effect of Temperature on PA-Induced Root Abscission

**Figure 4:**
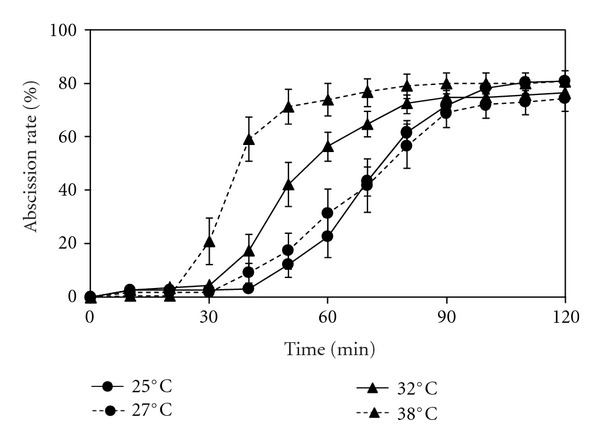
Effect of temperature gradient on spermine-induced root abscission in *A. pinnata*. Root abscission is represented as % of the initial number of roots following addition of 2 mM spermine at time zero. The assays were carried out at 25°C and 38°C (*n* = 6) and 27°C and 32°C (*n* = 9). Standard error bars are only shown where larger than the symbols used. Assays were conducted in 10 mM potassium phosphate solution (pH 7) at the time zero.

Exposure of *A. pinnata* to a temperature gradient did not affect the total abscission rate (75–80%) in response to Spm, but higher temperatures resulted in a significantly shorter lag time for the onset of abscission and total time required to reach 50% abscission rate (Figure 4). The time lag was 40 min at 25°C, 30 min at 27°C, and 32°C, 20 min at 38°C. Similarly to increasing temperature, the time required to shed 50% of the roots also decreased: 75 min at 25°C and 27°C, 60 min at 32°C, and 40 min at 38°C.

### 3.5. Effect of Light on PA-Induced Root Abscission. 

**Figure 5:**
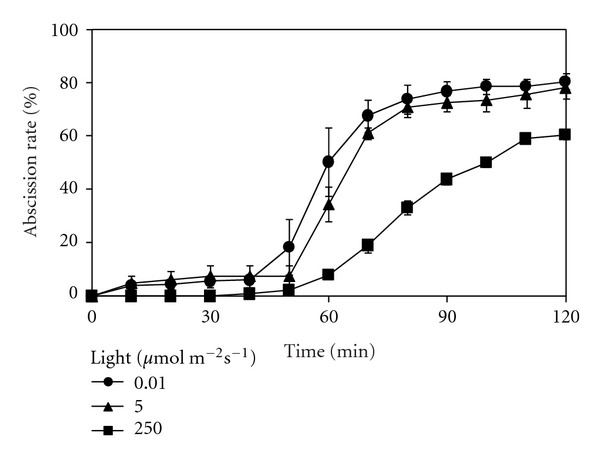
: Effect of light intensity on spermine-induced root abscission in *A. pinnata*. Root abscission is represented as % of the initial number of roots following addition of 2 mM spermine at time zero. Experiments were conducted under high light, 250 *μ*mol m^−2^ s^−1^ (*n* = 9); ambient light, 5 *μ*mol m^−2^ s^−1^ (*n* = 3) and dark condition, 0.01 *μ*mol m^−2^ s^−1^ (*n* = 6). Standard error bars are only shown where larger than the symbols used. Assays were conducted in 10 mM potassium phosphate solution (pH 7).

The availability of light as well as light intensity affected PAs-induced abscission rate, time required for the onset of abscission, and time to reach 50% root abscission in *A*. *pinnata *(Figure 5). Exposure to 2 mM Spm under ambient light in the laboratory showed a time lag of 50 min a total abscission rate of 80% and took 65 min for 50% root abscission. When the light intensity was increased by 50 times to 250 *μ*mol m^−2^ s^−1^, the time lag did not change but abscission rate decreased to 60%, and it took longer (100 min) to reach 50% root abscission. In contrast, dark conditions (0.01 *μ*mol m^−2^ s^−1^) stimulated the response; the time lag reduced to 40 min, 50% abscission was attained within 60 min, and final abscission rate at 2 h was 88%.

### 3.6. Effects of PA Oxidation Products on PA-Induced Root Abscission

**Figure 6:**
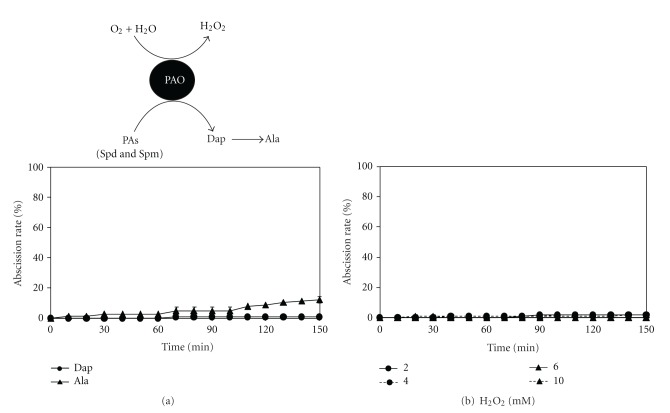
Effect of PA degradation products on root abscission in *A. pinnata*. The uppermost panel is the schematic representation of higher PAs oxidation by the enzyme polyamine oxidase (PAO) to yield 1,3-diaminopropane (Dap), *β*-alanine (Ala), and hydrogen peroxide (H_2_O_2_). Experiments in the presence of (a) 2 mM Dap (*n* = 3) or 2 mM Ala (*n* = 3) and (b) 2, 4, 6 and 10 mM H_2_O_2 _(*n* = 4). Standard error bars are only shown where larger than the symbols used. The experimental conditions were similar to those in Figure 2.

Previous studies have revealed an important role for PA catabolism via polyamine oxidase (PAO) in plant stress tolerance [21]. Thus, it was essential to check the effect of the PA oxidation products on the rapid root abscission phenomenon. The PA oxidation products Dap, Ala and H_2_O_2_ showed no significant abscission-triggering effect (Figures 6(a) and 6(b)). The abscission rates in the presence of Ala were only 12.5% and were negligible (<5%) in response to treatments with H_2_O_2_ and Dap. Furthermore, no change was observed in the total abscission rate by Spm in presence of hydroxyethyl hydrazine (HEH), an inhibitor of PAO (data not shown). These results suggest that Spm and Spd do not induce root abscission by supplying their degradation products; the parent compound Spm or Spd is required for the induction.

### 3.7. Effect of PAs on Nitrite-Dependent NO Production in *A. pinnata*

PA metabolism has been suggested to be associated with plant NO synthesis [23, 24, 27]. NO is a signaling molecule that is involved in diverse plant environmental responses [28, 29]. Possible crosstalk between PAs and NO in stress responses [18] led us to examine the involvement of NO in the PA-induced root abscission. 

**Figure 7:**
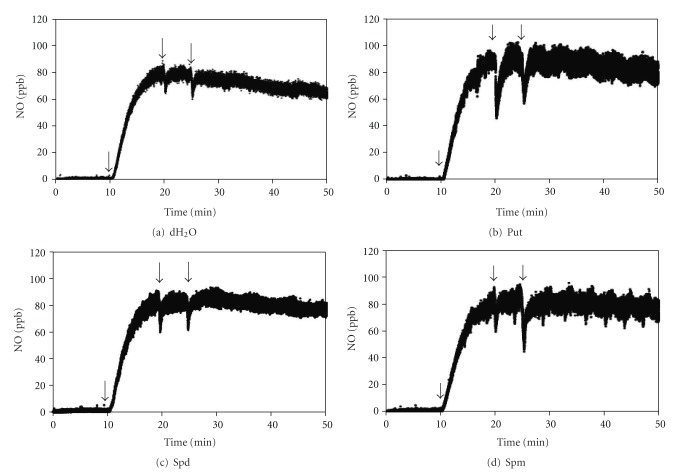
NO production by *A. pinnata* in response to addition of nitrite followed by polyamines. Confluent *A. pinnata* in 20 mL of 10 mM phosphate buffer at pH 7.0 in a 10 cm diameter petridish was supplemented with 0.1 mM nitrite at 10 min followed by addition of 400 *μ*L of dH_2_O (a), Put (b), Spd (c), and Spm (d) at 20 and 25 min. The arrows mark the additions of distilled water and polyamines.

NO released from *A. pinnata* was measured directly in the gas phase using a Sievers Nitric Oxide Analyzer (NOA) 280i (Figure 7). The basal NO production by *A. pinnata* was low (0–2.5 ppb). No detectable amount of NO signal was observed when the three major PAs were added (data not shown). However, when nitrite was supplied, the signal increased rapidly and reached to a range of 80–100 ppb. When dH_2_O or PAs were supplied, a slight decrease in the signal was observed for couple of seconds and the signal bounced back to its original pace of NO production. These results suggest that *A. pinnata* does not stimulate detectable NO emission in response to PAs and has no effect on nitrite-dependent NO production. 

## 4. Discussion

### 4.1. PAs as Inducers of Rapid Root Abscission

The presence of PAs in *A. pinnata* was found more than a decade ago [15] but their physiological roles have remained unclear. The present study has clearly shown that the PAs Spd and Spm are strong inducers of rapid root abscission whereas Put is ineffective in the short term (Figures 1 and 2). A smaller effect of Put relative to the higher PAs Spd and Spm has been found in many previous studies that investigated effects of PAs in various physiological processes, including stress responses [4, 7–9, 30]. Those different lines of observations may underlie an unknown common mechanism which involves Spd and Spm as inducers or signals for plant cells.

Assessment of the transport/uptake and metabolism of the exogenously added PAs could clarify the observed discrepancy among individual PAs. In higher plants, cellular uptake of PAs is very rapid (within a few minutes) and is known to be accelerated by auxin [31]. However, the identity of transporters for each polyamine remains unknown [23, 31]. Further studies on uptake, transport as well as measurement of cellular PAs on exposure to exogenous PAs are essential to address the abscission-inducing efficiency of PAs. 

### 4.2. Time Lag in the Effect of PAs

In the time courses of Spd or Spm-induced root abscission, the presence of a clear time lag to initiate abscission is interesting. A one-hour time lag should be beyond that required for the uptake of chemicals into cells (Figure 2). Since PAs are rapidly taken up by higher plants and stored in vacuoles [31], it is logical then to assume that the time lag reflects transport or their metabolism in the cells. Plants, including *A. pinnata*, contain the enzyme polyamine oxidase [32] which oxidizes higher PAs to yield H_2_O_2_, Dap, and Ala [21]. H_2_O_2_ is known to function as a signaling molecule in biotic and abiotic stress responses [28] while Ala derived from Dap functions as an osmoprotectant [33]. H_2_O_2_ produced by polyamine oxidase is also suggested to induce programmed cell death in the higher plant cells [34]. However, H_2_O_2_ could not stimulate abscission even at a very high concentration (Figure 4(b)). Furthermore, the other degradation products, Dap and Ala, also failed to trigger root abscission in *A. pinnata* (Figure 4(a)) which led us to rule them out as the candidate for the abscission inducer.

### 4.3. PAs and NO Production Pathway in *A. pinnata*

Tun and coworkers [23] and Silveira and coworkers [27] demonstrated that treatment with the PAs Spm and Spd results in the production of NO in higher plants, suggesting that NO may be involved in PA-mediated physiological response in plants. PAs are now considered as one of several candidate substrates for NO production in plants [35] via an unknown pathway [23, 24]. Laboratory cultures of *A*. *pinnata* cultivated under favorable conditions produced very low amounts of NO (Figure 7). Interestingly, nitrite, which stimulates NO production in *A. pinnata* (Figure 7), initiated rapid root abscission in *A*. *pinnata* with a shorter time lag compared with Spm or Spd [11]. The nitrite NO production pathway was unaffected by the three major PAs. Moreover, detectable amounts of NO emission from *A*. *pinnata* could not be measured in response to these PAs. Thus, our results do not point to an obvious role for NO in the polyamine-induced root abscission process in *A. pinnata.*

In conclusion our results have clearly shown that higher PAs Spd and Spm are efficient inducers of rapid root abscission in *A*. *pinnata*, and the subsequent rise in pH is not the only factor responsible for the onset of the abscission. To the best of our knowledge it is the first study to scrutinize the effect of PAs on the unique adaptive response of *A*. *pinnata*. Although PAs are omnipresent stress markers in plants exposed to virtually all stresses, their explicit mode of action remains largely unknown. The rapid root abscission phenomenon in *A*. *pinnata* provides a unique clue for understanding the function of PAs in stressed plant cells. 

## Abbreviations

Ala: *β*-alanine
Dap: 1, 3-diaminopropane
HEH: Hydroxyethyl hydrazine
H_2_O_2_: Hydrogen peroxide
NR: Nitrate reductase
NO: Nitric oxide
PAO: Polyamine oxidase
PAs: Polyamines
Put: Putrescine
SNN: Spermine NONOate
Spd: Spermidine
Spd·3HCl: Spermidine trihydrochloride
Spm: Spermine
Spm·4HCl: Spermine tetrahydrochloride.
